# Acute-onset Concomitant Esotropia in Children: A Comparison of Clinical Features and Treatment Outcomes

**DOI:** 10.18502/jovr.v20.14661

**Published:** 2025-07-30

**Authors:** Ying Wang, Jingjing Jiang, Li Li

**Affiliations:** ^1^Department of Ophthalmology, Beijing Children's Hospital, Capital Medical University, National Center for Children's Health, Key Laboratory of Major Diseases in Children, Ministry of Education, Beijing, China; ^2^Yangzhou Maternal and Child Health Care Hospital Affiliated to Yangzhou University, Yangzhou Women and Children's Hospital, Guangling District, Yangzhou, Jiangsu, China; ^3^These authors contributed equally to this work.

**Keywords:** Acute-onset Concomitant Esotropia, Botulinum Toxin A, Burian's Classifications, Clinical Features, Extraocular Muscle Surgery

## Abstract

**Purpose:**

To evaluate and compare the clinical features and efficacy of botulinum toxin A (BTX-A) injection versus surgery in acute-onset concomitant esotropia (ACE) in children.

**Methods:**

This retrospective comparative clinical study was conducted on 40 children with ACE. The patients were assigned to either the surgery group (*n* = 23) or the BTX-A group (*n* = 17). Successful motor outcomes were defined as residual esodeviation of < 5 prism diopters (PD), while successful sensory outcomes were defined as the presence of any evidence of sensory fusion or stereopsis.

**Results:**

The average treatment age in this study was 7.02 ± 3.72 years in the BTX-A group and 6.41 ± 2.83 years in the surgery group. There were 5 cases of type I (12.50%), 26 cases of type II (65.00%), and 9 cases of type III (22.50%) for ACE. The deviation before treatment was +(41.35 ± 16.90) PD at near and +(39.71 ± 14.94) PD at distance in the BTX-A group. In the surgery group, the results were +(49.22 ± 18.25) PD at near and +(47.00 ± 18.53) PD at distance. After treatment, based on the measured deviation, total motor success was 95.00% (38/40) at near and 90.00% (36/40) at distance. Following treatment, 94.59% (35/37) of patients with ACE exhibited fusion, 86.84% (33/38) had near stereopsis, and 72.97% (27/37) had distance stereopsis. The motor and sensory success rates were not significantly different between the BTX-A and surgery groups. There were no statistically significant differences in motor outcomes or sensory outcomes among the three subtypes of ACE (all *P* > 0.05).

**Conclusion:**

In our study, type II was the most common clinical classification for ACE. Favorable outcomes could be achieved with both BTX-A injection and surgery. There was no difference in motor or sensory outcomes regardless of clinical classification. BTX-A injection is minimally invasive, allows early intervention, and may be the preferred approach for managing ACE in children.

##  INTRODUCTION

Acute-onset concomitant esotropia (ACE) is a peculiar type of esotropia characterized by the sudden onset of a concomitant esotropia with horizontal diplopia.^[1]^ ACE usually occurs in older children and adults who have developed complete binocular visual function. In 1958, Burian and Miller divided ACE into three clinical subtypes according to its pathogenesis and etiology: Type I (Swan type), which is associated with the therapeutic patching of one eye, occlusion of the optic axis, or loss of vision in one eye; Type II (Franceschetti type), which is characterized by a large esodeviation with a mild degree of hyperopia; and Type III (Bielschowsky type), which usually occurs in older children and adults with uncorrected myopia ≥ –5.00 diopter (D).^[2]^ In recent years, the prevalence of ACE has increased significantly, and the age of onset has also decreased.^[3]^ Some researchers have suggested that the main reason for this is the excessive use of smartphones or other electronic devices by adolescents.^[4]^ However, the number of children with ACE is also increasing, and the associated risk factors and clinical features are still not clear.

The methods used to treat ACE include prism therapy, extraocular muscle surgery, and botulinum toxin A (BTX-A) injection.^[5]^ However, no standard treatment modality or protocol has been established, although surgery was the preferred approach for managing ACE in the past. Among ophthalmologists who recommend surgery, many prefer to wait until the angle of deviation is stable to increase the likelihood of a successful outcome. In contrast, others perform the surgical intervention as soon as possible, arguing that waiting for the patient to reach an older age or a stable deviation tends to result in missing the best opportunity for treatment and a prolonged period of diplopia.^[6]^ Recently, an increasing number of ophthalmologists have adopted BTX-A injection as an early intervention method for ACE and have reported good outcomes.^[7]^

BTX-A is a neurotoxin that causes muscle paralysis.^[8]^ In 1981, Scott first reported the use of BTX-A for treating strabismus,^[9]^ and it has been used in various types of strabismus treatment ever since.^[10,11,12]^ BTX-A injection has several unique advantages; for example, it is a minimally invasive method, requires a shorter treatment period, and allows for earlier intervention.^[13,14]^ Previous studies have shown that BTX-A injection and surgery are equally effective treatment methods for ACE.^[15,16]^

However, the use of BTX-A for ACE in children has not been explored in these studies. It is well-known that children with esotropia are more likely to experience suppression than adults with stable binocular vision. Therefore, given this gap in the literature and the fact that the number of children with ACE has steadily increased in recent years,^[17]^ there is a need to further investigate the treatment of ACE in children.

This study aimed to evaluate and compare the clinical features and treatment outcomes of BTX-A injection and surgery for ACE in children to determine which approach offers the best results for the three types of ACE.

##  METHODS

We enrolled children with ACE who underwent treatment in Beijing Children's Hospital and were followed up for at least six months between January 2020 and January 2022.

Participants comprised patients with ACE who presented with a sudden onset, diplopia, and no limitation of eye movement, were aged 3 to 18 years old, and could be followed for six months or more. We excluded patients with vertical strabismus or other types of strabismus, amblyopia or other functional eye diseases, previous BTX-A injections or extraocular muscle surgery, history of head trauma, and any other ophthalmic, systemic, or neurological condition that could affect ocular alignment and visual function.

This was a retrospective, non-randomized, comparative clinical study that did not require obtaining informed consent from the patients. This research was approved by the Ethics Committee of Beijing Children's Hospital of Capital Medical University of China (No. 2020-k-171) and was registered on the Chinese Clinical Trial Registry (http://www.chictr.org.cn; ChiCTR-INR-17013777). This research adhered to the principles outlined in the Declaration of Helsinki.

All patients underwent routine ophthalmic examinations, including slit lamp to check the anterior segment of the eye, dilated fundus examination, best corrected visual acuity (BCVA), and cycloplegic refraction. We administered 1% atropine eye gel daily for three consecutive days before the refractive assessment. Objective refraction was performed by retinoscope and auto refractometer (ARK-1 Auto Refractometer, NIDEK, Japan). Subjective refraction was done after objective refraction if patients could cooperate. The alternate prism and cover test was used to measure the angle of deviation at distance (6 m) and near (33 cm) if glasses were prescribed for full refractive correction. Diplopia was evaluated by the red glass filter test. Fusion and stereopsis at distance fixation were documented by synoptophore testing, whereas stereopsis at near fixation was assessed with the Titmus stereo test. Neuroimaging was done to rule out intracranial pathology. We collected data on several parameters, including the onset of the esotropia, the duration of esotropia, prior treatment for strabismus, systemic comorbidities, and family history. Patients were analyzed based on the subtype of ACE present.

The treatment methods were determined based on the preference of children and their guardians. The patients were classified into two groups based on their treatment method: the surgery group and the BTX-A group. All procedures were performed under general anesthesia. In the cases of BTX-A injection (HengLi, Lanzhou Institute of Biological Products Co, China), the children were treated with bilateral injections of BTX-A into the medial rectus muscles according to the preoperative angle of deviation: patients with an angle of deviation of < 50 prism diopters (PD) received 2.5 IU/0.1 mL BTX-A. Those with an angle of deviation of > 50 PD received 5.0 IU/0.1 mL BTX-A. Surgical procedures consisted of bilateral or unilateral medial rectus muscle recession or lateral rectus muscle resection.

The angle of deviation, fusion, and stereopsis of each patient were recorded before and after treatment. The successful motor outcome was defined as residual esodeviation < 5 PD. Sensory success was defined as the presence of any evidence of sensory fusion and stereopsis, along with the absence of any diplopia at the last visit. The effective rate and proportion of children with fusion and stereopsis were compared between the two groups. Before treatment, we evaluated the clinical features associated with the three subtypes of ACE, including age of onset, spherical equivalent (SE), and angle of deviation at near and distance. After treatment, the motor and sensory outcomes were also compared across the three subtypes. During the follow-up period, we also recorded different complications such as penetrating eye injury, ptosis, vertical strabismus, slippage of muscles, overcorrection, subconjunctival hemorrhage, conjunctival cyst, and eye movement disorders after surgery or BTX-A injection.

Statistical analysis was performed by SPSS 25.0. The data involved in this research were tested for normality of continuous variables, and the results showed a normal distribution; therefore, they were presented in terms of mean ± standard deviation (X ± S). Statistical analysis included an independent samples *t*-test and analysis of variance (ANOVA). Other variables were presented as medians and interquartile ranges (*M *[*Q* ${}_{1}$ , *Q* ${}_{3}$ ]) and analyzed using the Mann–Whitney U test. Categorical variables were expressed as frequencies and percentages, and comparisons were performed using the chi-square ( χ ^2^) test. *P* < 0.05 was considered statistically significant.

**Table 1:** Basic clinical features

**Characteristic**	**BTX-A group**	**Surgery group**	*t*/**χ^2^**	*P*-**value**
Age (years)	7.02 ± 3.72 (3.6 to 15.8)	6.41 ± 2.83 (3.3 to 14.9)	0.595	0.556
Sex (*n*, %) Male Female	11 (64.71%) 6 (35.29%)	15 (65.22%) 8 (34.78%)	0.001	0.973
Duration of esotropia (month)	4.18 ± 5.05 (1 to 18)	9.57 ± 7.38 (2 to 36)	–2.590	0.014
Spherical equivalent (D)				
Right eye	–0.01 ± 2.42 (–7.00 to +2.375)	+0.99 ± 2.01 (–4.00 to +6.00)	–1.432	0.160
Left eye	+0.16 ± 2.33 (–7.00 to +2.50)	+0.99 ± 1.76 (–2.50 to +4.375)	–1.278	0.209
Best corrected visual acuity				
Right eye	0.89 ± 0.15 (0.6 to 1.0)	0.90 ± 0.13 (0.6 to 1.0)	–0.268	0.790
Left eye	0.90 ± 0.13 (0.6 to 1.0)	0.92 ± 0.11 (0.7 to 1.0)	–0.571	0.572
Preoperative angle of deviation (PD)				
Near (33 cm)	+41.35 ± 16.90 (+8 to +70)	+49.22 ± 18.25 (+25 to +105)	–1.390	0.173
Distance (6 m)	+39.71 ± 14.94 (+10 to +65)	+47.00 ± 18.53 (+20 to +105)	–1.332	0.191
The "+" in the preoperative angle of deviation indicates the esodeviation. BTX-A, botulinum toxin A; cm, centimeter; D, diopters; m, meter; PD, prism diopters				

##  RESULTS

A total of 40 patients were enrolled: 23 in the surgery group and 17 in the BTX-A group. There was no statistically significant difference in gender, age, SE, BCVA, or preoperative angle of deviation at near and distance between the two groups (all *P* > 0.05). The mean duration of esotropia was 9.57 ± 7.38 months in the surgery group, which was significantly longer than that in the BTX-A group (4.18 ± 5.05 months; *P *= 0.014). The basic clinical features of the study participants, classified by treatment group, are shown in Table 1.

Reviewing each patient's medical history and clinical features, we identified 5 cases of ACE Type I (12.50%), 26 cases of ACE Type II (65.00%), and 9 cases of ACE Type III (22.50%).

There was no significant difference in the baseline angle of deviation at near and distance across the three clinical classifications of ACE (near: *F *= 0.330, *P *= 0.721; distance: *F *= 0.532, *P *= 0.592). There was also no significant difference between near and distance values for each type of ACE (Type I: *t *= 0.185, *P *= 0.858; Type II: *t *= 0.762, *P *= 0.450; Type III: *t *= 0.024, *P *= 0.981). The mean age of patients with Type III was 11.12 ± 3.62 years, which was older than that of patients with the other two types (Type I: 5.40 ± 1.71 years, *P *= 0.004; Type II: 5.37 ± 1.47 years, *P *= 0.005). The mean SE in both eyes of the patients with Type III indicated a higher myopia than the other two types (Right eye: –2.47 ± 2.23 D; Left eye: –2.06 ± 2.36 D), whereas the refractive data indicated patients in Type I and Type II had minor hyperopia (*P *= 0.007, *P *= 0.003; *P *= 0.004, *P *= 0.008), and there was no statistically significant difference between the patients with Type I and Type II (*P *= 0.587, *P *= 0.597). The data are shown in Table 2.

When the data for the angle of deviation after treatment were analyzed, it was found that the success rate was 95.00% (38/40) at near and 90.00% (36/40) at distance in all patients with ACE. At near and distance, motor success rates were 94.12% (16/17) and 88.24% (15/17) in the BTX-A group and 95.65% (22/23) and 91.30% (21/23) in the surgery group, respectively. There was no statistically significant difference between the two groups at either distance ( $\chi {}^{2}$ = 0.049, *P *= 0.826) [Table 3].

**Figure 1:**
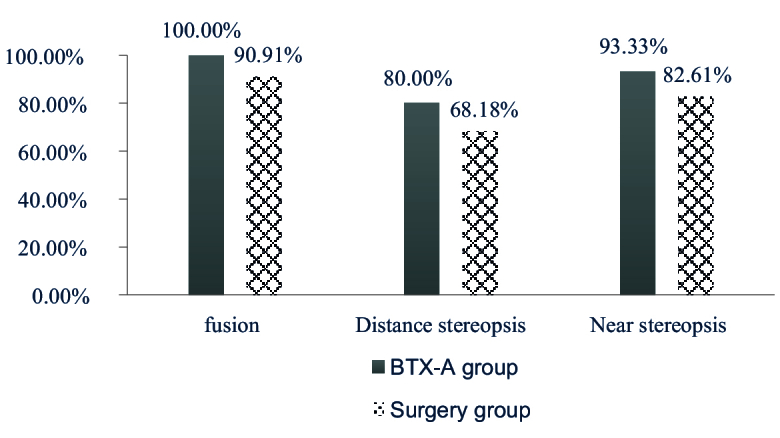
The sensory outcomes in the BTX-A and surgery groups.

**Figure 2:**
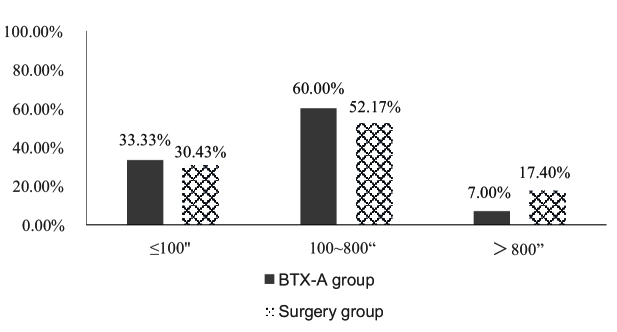
Quantitative evaluation of near stereopsis in the BTX-A and surgery groups.

Two children in the BTX-A group and one child in the surgery group were uncooperative during the fusion and stereopsis examination. After treatment, 94.59% (35/37) of the patients exhibited fusion, while 86.84% (33/38) and 72.97% (27/37) showed near and distance stereopsis, respectively. Specifically, fusion developed in all patients (15/15) in the BTX-A group and 90.91% (20/22) of patients in the surgery group, with no statistically significant difference between the two groups ( χ ${}^{2}$ = 1.442, *P *= 0.230). Additionally, 93.33% (14/15) and 80.00% (12/15) of patients in the BTX-A group had near and distance stereopsis, respectively, and 82.61% (19/23) and 68.18% (15/22) of patients in the surgery group had near and distance stereopsis, respectively. There was no significant difference between the two groups ( χ ^2^ = 0.914, *P *= 0.339; χ ^2^ = 0.632, *P *= 0.427), as shown in Table 3 and Figure 1. It was found that 33.33% (5/15) of patients in the BTX-A group and 30.43% (7/23) of those in the surgery group achieved stereopsis better than 100", and the two groups showed no difference in terms of stereopsis in the quantitative analysis ( χ ^2^ = 0.918, *P *= 0.632) [Table 3; Figure 2].

Motor success rates, based on the angle of deviation at near and distance, were as follows: Type I: 100.00% (5/5) at near and 80.00% (4/5) at distance. Type II: 96.15% (25/26) for both near and distance. Type III: 88.89% (8/9) at near and 77.78% (7/9) at distance. Statistical analysis showed no significant difference in motor success rates across the three types of ACE (near: χ ^2^ = 1.450, *P *= 0.583; distance: χ ^2^ = 3.708, *P *= 0.150). The sensory success rate was 80.00% (4/5) for Type I, 84.62% (22/26) for Type II, and 100.00% (9/9) for Type III, and there were no statistically significant differences among the three types ( χ ^2^ = 1.724, *P *= 0.475) [Table 4].

In the BTX-A group, 35.29% (6/17) of the patients developed complications; there were three cases of unilateral ptosis with overcorrection, two cases of vertical strabismus, and one case of subconjunctival hemorrhage. However, the complications were temporary in all six cases and disappeared within three months. In the surgery group, 21.74% (5/23) of the patients developed complications. In this group, we observed three cases of overcorrection, one case of an eye movement disorder, and one case of a conjunctival cyst. The incidence of complications did not significantly differ between the two groups ( χ ^2^ = 0.901, *P *= 0.276).

**Table 2:** Patients' clinical features according to the Burian's classification of ACE

	**Type I**	**Type II**	**Type III**
Age (years)	5.40 ± 1.71	5.37 ± 1.47	11.12 ± 3.62
F ${}^{*}$ ,* P*	24.935, 0.000		
Spherical equivalent refractive error (D)			
Right eye	+2.48 ± 2.06	+1.25 ± 0.94	–2.47 ± 2.23
F ${}^{*}$ , *P*	26.492, 0.000		
Left eye	+2.13 ± 1.41	+1.28 ± 0.95	–2.06 ± 2.36
F ${}^{*}$ ,* P*	21.457, 0.000		
Baseline angle of deviation (PD)			
Near (33 cm)	51.40 ± 16.88	44.42 ± 12.38	47.00 ± 30.22
F ${}^{*}$ ,* P*	0.330, 0.721		
Distance (6 m)	49.40 ± 17.30	41.88 ± 11.63	46.67 ± 29.05
F ${}^{*}$ ,* P*	0.532, 0.592		
*t* ${}^{*}$	0.185	0.762	0.024
*P*	0.858	0.450	0.981
ACE, acute-onset concomitant esotropia; cm, centimeter; m, meter; D, diopters; PD, prism diopters ${}^{*}$ An ANOVA was performed to compare the differences in age, spherical equivalent refractive error, and baseline angle of deviation among the three types of ACE. The *t*-test was used to analyze the difference in the angle of deviation between near and distance conditions for each subtype.			

**Table 3:** Comparison of motor and sensory outcomes in the BTX-A and surgery groups

	**BTX-A group**	**Surgery group**	**Total**	*t*/**χ^2^**	*P*-**value**
Angle of deviation at near (33 cm)	94.12% (16/17)	95.65% (22/23)	95.00% (38/40)	0.049	0.826
Angle of deviation at distance (6 m)	88.24% (15/17)	91.30% (21/23)	90.00% (36/40)	0.102	0.749
Fusion	100.00% (15/15)	90.91% (20/22)	94.59% (35/37)	1.442	0.230
Distance stereopsis	80.00% (12/15)	68.18% (15/22)	72.97% (27/37)	0.632	0.427
Near stereopsis	93.33% (14/15)	82.61% (19/23)	86.84% (33/38)	0.914	0.339
Near stereopsis better than 100"	33.33% (5/15)	30.43% (7/23)	31.58% (12/38)	0.918	0.632
", arc seconds; BTX-A, botulinum toxin A; cm, centimeter; m, meter					

**Table 4:** Comparison of motor and sensory outcomes among patients with different Burian's classifications of ACE

	**Type I**	**Type II**	**Type III**	*t*/**χ^2^**	*P*-*value*
Motor outcome					
Angle of deviation at 33 cm	100.00% (5/5)	96.15% (25/26)	88.89% (8/9)	1.450	0.583
Angle of deviation at 6 m	80.00% (4/5)	96.15% (25/26)	77.78% (7/9)	3.708	0.150
Sensory outcome	80.00% (4/5)	84.62% (22/26)	100.00% (9/9)	1.724	0.475
ACE, acute-onset concomitant esotropia; cm, centimeter; m, meter					

##  DISCUSSION

The etiology of ACE is complex and involves a variety of factors, including fusion decompensation, intracranial pathology, myopia, a history of occlusion, and monocular fixation syndrome. Physical and mental factors also play a role in its development.^[18]^ Most of the published studies on ACE have involved adults and children, and according to the clinical types defined by Burian, the incidence of Type III is relatively high in adults.^[19]^ Sefi-Yurdakul analyzed 13 children and found 11 cases (84.6%) of ACE had been caused by excessive close work on computer and smartphone screens.^[20]^ In contrast to previous studies that introduced Type III as the main subtype, we found Type II as the most common, accounting for 65.00% of ACE cases in the children who were studied.^[21]^ This raises the question of whether the frequency of ACE in this cohort is influenced by environmental factors versus race and genetics, or both. Hence, there is a need for further research on ACE in children.

As a treatment for strabismus, BTX-A is applied to temporarily paralyze the injected extraocular muscles, thereby increasing the strength of the antagonist muscles. It is expected that the extraocular muscles will then adjust to achieve ocular alignment. Wan et al compared the effects of BTX-A injection with those of surgery in children with ACE. The results showed that the success rates recorded in their groups six months after the treatment were not significantly different (81% vs 61%).^[22]^ In this study, the success rates were 94.12% at near and 88.24% at distance in the BTX-A group and 95.65% at near and 91.30% at distance in the surgery group. Our results suggest that BTX-A injection is as effective as surgery in managing ACE. This aligns with some previous studies indicating that both surgery and BTX-A injection for ACE can alleviate strabismus and diplopia.^[22,23]^

For children with ACE, BTX-A injection offers several unique advantages. First, it is feasible to inject BTX-A when ACE is in an early stage and the angle of deviation is not stable.^[24]^ This may help reduce deterioration of ocular alignment and loss of binocular function. Second, the extraocular muscles remain intact after BTX-A injection, unlike in surgery.^[25]^ In other words, this injection does not alter the extraocular muscle's anatomical position or result in scarring that may affect function or future interventions. Third, the procedure for BTX-A injection is simple and relatively brief, significantly reducing anesthesia time for children.^[25]^ It is also worth mentioning that BTX-A injection costs less than surgery.^[15]^

Many patients with ACE have fully developed binocular visual function before the onset of the disease. It has been shown that stereopsis can be easily restored after treatment.^[15]^ In this study, 93.33% and 80.00% of patients in the BTX-A group and 82.61% and 68.18% of patients in the surgery group recovered normal stereopsis at near and distance, respectively. It should be noted that three patients were excluded from the visual function examination because they were younger than four years old and therefore did not undergo this part of the analysis. In Huang's study, only 3 out of 33 patients had no stereoscopic indication after treatment,^[26]^ a finding that aligns with our results.

Patients with ACE generally present with a short duration of disease and no suppression of the strabismic eye; therefore, the damage to their binocular visual function is mild. When treated promptly, the visual function tends to recover well after the eye alignment is corrected.^[26]^ In this study, two patients in the BTX-A group and seven patients in the surgery group did not obtain stereopsis after treatment. This was likely because they were under four years old and had not yet developed binocular vision before the onset of the disease, in addition to the fact that the disease had been ongoing for longer than 12 months.

Generally, both BTX-A injection and surgery proved to be effective ways of treating ACE in our study. There was no difference in the motor and sensory outcomes across the three types of ACE, indicating that the efficacy of treatment was the same regardless of the clinical classification.

This research had some limitations. For example, the study was retrospective and non-randomized, the sample size was small, and the follow-up was limited. Thus, further prospective studies should be conducted to determine the optimal treatment for ACE in children.

In summary, Franceschetti Type II was the most common subtype of ACE in this study. There was no difference in motor or sensory outcomes regardless of clinical classification. Good outcomes were achieved in almost all patients receiving either BTX-A injection or extraocular muscle surgery, with no difference in the efficacy of the treatment methods across the three clinical subtypes. Our findings also indicated that BTX-A injection is a minimally invasive procedure, requires shorter anesthesia time, and allows for early intervention.

##  Financial Support and Sponsorship

This research was funded by the National Natural Science Foundation of China (No. 82371093), the R&D Program of Beijing Municipal Education Commission (No. KZ202110025039), and the Capital's Funds for Health Improvement and Research (No. 2022-1G-4251).

##  Conflicts of Interest

None.
